# *Anaerostipes hadrus*, a butyrate-producing bacterium capable of metabolizing 5-fluorouracil

**DOI:** 10.1128/msphere.00816-23

**Published:** 2024-03-12

**Authors:** Danping Liu, Li-Sheng Xie, Shitao Lian, Kexin Li, Yun Yang, Wen-Zhao Wang, Songnian Hu, Shuang-Jiang Liu, Chang Liu, Zilong He

**Affiliations:** 1 School of Engineering Medicine, Beihang University, Beijing, China; 2 Key Laboratory of Big Data-Based Precision Medicine, Beihang University, Ministry of Industry and Information Technology of the People’s Republic of China, Beijing, China; 3 Key Laboratory of Biomechanics and Mechanobiology, Beihang University, Ministry of Education, Beijing, China; 4 State Key Laboratory of Microbial Technology, Shandong University, Qingdao, China; 5 Systems Biology and Bioinformatics (SBI), Leibniz Institute for Natural Product Research and Infection Biology-Hans Knöll Institute (HKI), Jena, Germany; 6 State Key Laboratory of Mycology, Institute of Microbiology, Chinese Academy of Sciences, Beijing, China; 7 State Key Laboratory of Microbial Resources, Institute of Microbiology, Chinese Academy of Sciences, Beijing, China; National Institute of Advanced Industrial Science and Technology, Tsukuba, Ibaraki, Japan

**Keywords:** *Anaerostipes hadrus*, butyrate, 5-fluorouracil, pan-genomics, metagenomics

## Abstract

*Anaerostipes hadrus* (*A. hadrus*) is a dominant species in the human gut microbiota and considered a beneficial bacterium for producing probiotic butyrate. However, recent studies have suggested that *A. hadrus* may negatively affect the host through synthesizing fatty acid and metabolizing the anticancer drug 5-fluorouracil, indicating that the impact of *A. hadrus* is complex and unclear. Therefore, comprehensive genomic studies on *A. hadrus* need to be performed. We integrated 527 high-quality public *A. hadrus* genomes and five distinct metagenomic cohorts. We analyzed these data using the approaches of comparative genomics, metagenomics, and protein structure prediction. We also performed validations with culture-based *in vitro* assays. We constructed the first large-scale pan-genome of *A. hadrus* (*n* = 527) and identified 5-fluorouracil metabolism genes as ubiquitous in *A. hadrus* genomes as butyrate-producing genes. Metagenomic analysis revealed the wide and stable distribution of *A. hadrus* in healthy individuals, patients with inflammatory bowel disease, and patients with colorectal cancer, with healthy individuals carrying more *A. hadrus*. The predicted high-quality protein structure indicated that *A. hadrus* might metabolize 5-fluorouracil by producing bacterial dihydropyrimidine dehydrogenase (encoded by the *preTA* operon). Through *in vitro* assays, we validated the short-chain fatty acid production and 5-fluorouracil metabolism abilities of *A. hadrus*. We observed for the first time that *A. hadrus* can convert 5-fluorouracil to α-fluoro-β-ureidopropionic acid, which may result from the combined action of the *preTA* operon and adjacent *hydA* (encoding bacterial dihydropyrimidinase). Our results offer novel understandings of *A. hadrus*, exceptionally functional features, and potential applications.

**IMPORTANCE:**

This work provides new insights into the evolutionary relationships, functional characteristics, prevalence, and potential applications of *Anaerostipes hadrus*.

## INTRODUCTION

Trillions of microorganisms in the human gut are diverse and functionally rich. Species belonging to *Anaerostipes*, *Eubacterium*, *Faecalibacterium*, *Clostridium*, and other genera can produce butyrate through gastrointestinal bacterial fermentation (1). Butyrate is essential for maintaining intestinal epithelial cell barrier function, regulating the immune response of intestinal mucosa, and preventing cancer (2). The decrease in butyrate-producing species is closely associated with various diseases such as ulcerative colitis (UC) (3), Crohn’s disease (CD) (4), intestinal lymphoma (5), and type 2 diabetes (6). However, not all butyrate-producing bacteria have positive effects on the host. In addition to toxigenic strains of *Clostridium butyricum* that can cause botulism and necrotizing enterocolitis (7), *Anaerostipes hadrus* (*A. hadrus*) is also noteworthy as the first butyrate-producing species shown harmful effects on host health under certain disease-inducing conditions (8).

*A. hadrus* is a representative commensal bacterium with a relative abundance of 2%–7% in human intestines (9, 10). Previous studies have confirmed that *A. hadrus* can produce high levels of butyrate from sugars or acetate and lactate metabolized by other bacteria (11, 12). In addition, *A. hadrus* can also metabolize fructooligosaccharides to support the growth of other bacteria, such as *Lacticaseibacillus rhamnosus* (13). Furthermore, researchers identified that *A. hadrus* possesses biotin synthesis genes that regulate immunity and inflammation (14). Therefore, some scholars consider *A. hadrus* a beneficial bacterium (15). Consuming either milk (14) or galactooligosaccharides (16) can increase the abundance of *A. hadrus* in the gut microbiota. Nevertheless, a recent multi-omics study indicated that *A. hadrus*-mediated fatty acid biosynthesis influenced the availability of long-chain free fatty acids in the portal circulation and enhanced hepatic fibrosis (17). Another study mentioned that *A. hadrus* could inactivate the anticancer drug 5-fluorouracil (5-FU) (18), but the metabolic mechanism and pathway underlying this process still need to be better understood. The evidence above indicates that the precise role of *A. hadrus* in maintaining human health remains unclear. Thus, further culture independent and -dependent on *A. hadrus* is necessary.

With the advancement in microbiome research, metagenomics and culturomics have gradually become essential tools for studying microbes (19, 20). In this study, we completed the first large-scale population analysis of *A. hadrus* by integrating public genomic data. Moreover, we first characterized the ability of *A. hadrus* to convert 5-FU into α-fluoro-β-ureidopropionic acid (FUPA) as a dead-end metabolite using both *in silico* identifications of target genes and culture-based biotransformation assays. This work provides new insights into the evolutionary relationships, functional characteristics, prevalence, and potential applications of *A. hadrus*.

## MATERIALS AND METHODS

### Acquisition and quality control of public *A. hadrus* genomes

The overall analysis workflow is described in Fig. S1. First, we downloaded all *A. hadrus* genomes from GenBank (21) (https://ftp.ncbi.nlm.nih.gov/genomes/genbank/bacteria/Anaerostipes_hadrus/) and the Unified Human Gastrointestinal Genome [UHGG (22), http://ftp.ebi.ac.uk/pub/databases/metagenomics/mgnify_genomes/human-gut/v1.0/] collection, including isolate genomes and metagenome-assembled genomes (MAGs). Second, we used Kraken (23) (version 2.1.2) and Bracken (24) (version 2.6.1) to ensure the taxonomic classification. Then, we also used QUAST (25) (version 5.2.0) and BUSCO (26) (version 5.4.3) to assess the genome quality and core gene content, respectively. Finally, we applied CheckM (27) (version 1.2.2) to determine the genome completeness and contamination. Genomes analyzed in this study were required to have >90% completeness (CheckM), <5% contamination (CheckM), and >90% core genes (BUSCO). In addition, a size between 2.8 and 3.4 Mbp was required for isolate genomes. For MAGs, it should be between 2.5 and 3.4 Mbp. All software used default parameters during the analysis. More information about *A. hadrus* genomes can be found in Tables S1 and S2.

### Pan-genome construction

We performed the gene annotation of screened *A. hadrus* genomes with Prokka (28) (version 1.14.6). Based on the annotation result (.gff file), we constructed the *A. hadrus* pan-genome with Roary (29) (version 3.10.2). Both Prokka and Roary used default parameters.

### Phylogenetic and functional annotation analysis

According to the core gene alignment result generated by Roary, we constructed the phylogenetic tree of *A. hadrus* genomes using FastTree (30) (version 2.1.10) with the following parameters: "-nt -gtr". The average nucleotide identity (ANI) between *A. hadrus* genomes was calculated by Pyani (31) (version 0.2.12) with the parameter: "-m ANIm." We also constructed the multispecies phylogenetic tree of the genus *Anaerostipes* based on PhyloPhlAn (32) (version 3.0.67) and RAxML (33) (version 8.2.12). PhyloPhlAn used the parameters "--diversity low --fast -d phylophlan" and RAxML used the parameters "-f a -x 12,345 p 12345 -# 1000 m PROTGAMMAAUTO." The phylogenetic tree was visualized using ggtree (34) (version 3.2.1). Functional differences of representative genomes from different *A. hadrus* evolutionary clades were analyzed with the KEGG Automatic Annotation Server [KAAS (35), https://www.genome.jp/tools/kaas/]. Basic information of representative genomes for constructing the phylogenetic tree of the genus *Anaerostipes* is detailed in Table S3.

### Analysis of butyrate-producing genes and 5-FU metabolism genes

We first selected reference protein sequences of butyrate-producing genes and 5-FU metabolism genes from Swiss-Prot as queries to perform tblastn (36) (version 2.13.0+) alignment with *A. hadrus* genomes (screening parameters: identity ≥30%, e-value <1e−10, query coverage ≥90%). Next, we verified alignment results with the non-redundant protein sequence database (NR) using blastx (36) (version 2.13.0+) alignment (screening parameters: identity ≥90%, query coverage ≥90%, subject coverage ≥90%, e-value <1e−10) and finally determined the sequences of target genes in *A. hadrus* genomes. Furthermore, we identified the upstream and downstream genes of butyrate-producing genes and 5-FU metabolism genes based on genome annotation files generated by Prokka. These gene sequences annotated by Prokka were also verified through the blastp [DIAMOND (37) version 2.0.15.153] alignment (screening parameters: identity ≥99%, e-value <1e−10) against NR. Finally, we used MEME Suite (38) (https://meme-suite.org/meme/meme_5.5.0/) to predict possible motifs lying upstream of 5-FU metabolism genes. The protein structure of *A. hadrus* PreT-PreA heterodimer was predicted by ColabFold (39) (https://colab.research.google.com/github/sokrypton/ColabFold/blob/v1.3.0/AlphaFold2.ipynb) with the following parameters: use_templates, true; use_amber, true; msa_mode, MMseqs2 (UniRef +Environmental); model_type, AlphaFold2-multimer-v2; num_models, 5; num_recycles, 6; pair_mode: unpaired +paired. The known protein structure was downloaded from the protein data bank (PDB). The alignment and visualization of protein structures were performed using PyMOL (http://www.pymol.org/pymol) (version 2.6.0a0).

### Antibiotic resistance genes and virulence factor identification

To identify antibiotic resistance genes from CARD (version 3.2.6) in *A. hadrus* genomes, we analyzed the amino acid sequences of *A. hadrus* genomes using the Resistance Gene Identifier [RGI (40), version 4.0.3]. Genes identified by the Perfect algorithm in RGI were curated antibiotic resistance genes in CARD, while genes identified by RGI using the Strict algorithm were considered potential antibiotic resistance genes and required validation through comparison to NR. To investigate virulence factors in *A. hadrus* genomes, we performed a blastp (DIAMOND version 2.0.15.153) alignment between amino acid sequences of *A. hadrus* genomes and VFDB with filtering parameters of identity ≥60% and subject coverage ≥80%.

### Determination of short-chain fatty acids

The concentrations of short-chain fatty acids (SCFAs, including acetate, propionate, butyrate, valerate, isobutyrate, and isovalerate) were determined using gas chromatography-mass spectrometry (GC-MS) as described in our previous research (9). In brief, the *Anaerostipes hadrus* CGMCC 1.32965 was incubated at 37°C anaerobically in modified mGAM broth for 72 h. Then, 1 mL of cell culture was extracted with 1 mL of ethyl acetate, and the supernatant was prepared for GC-MS analysis performed on a GCMS-QP2010 Ultra with an auto-sampler (SHIMADZU, Japan) and the DB-wax capillary column (30 m, 0.25 mm i.d., 0.25-µm film thickness, SHIMADZU, Japan). Standard curves of SCFAs were achieved by pure chemical agents of corresponding chemicals, purchased from Aladdin (Shanghai, China). The temperature of the oven was programmed from 35°C to 130°C at 5°C/min gradients, to 230°C at 30°C/min gradients, with a 16-min hold. Injection of 2 µL of samples was performed at 230°C. The carrier gas, helium, flowed at 1.0 mL/min. Ion source and interface temperature were both set at 230°C. The electronic impact was recorded at 70 eV.

### Determination of 5-fluorouracil and its metabolites

To determine the degradation of 5-fluorouracil (5-FU) or production of α-fluoro-β-ureidopropionic acid (FUPA) by *Anaerostipes hadrus* cells *in vitro*, the *Anaerostipes hadrus* was incubated in modified MMGMB media for over 24 h until the microbes reached the stationary phase. The cells were harvested by centrifugation and washed with PBS buffer in anerobic chamber. After cell counting under microscopy, proper volume of resuspended cell solution was added to a 10-mL reaction system containing 5 mM of 5-FU at a final concentration of 10^9^ cells/mL. The reaction system was incubated at 37°C under anerobic condition and at time points 0, 0.5, 1, 2, 3, and 6 h. A 1-mL reaction solution was sampled and centrifugated. The supernatant was used to analyze the concentration of 5-FU and FUPA with an Agilent Accurate-Mass-Q-TOF LC/MS 6520B instrument (Agilent, Germany) as described below: A Shim-pack GIST C18-AQ column (250 mm × 4.6 mm i.d.; 5 µm; SHIMADZU, Japan) was used at 35°C with a flow rate of 0.8 mL/min for liquid chromatography separation. The injection volume was 2 µL. The mobile phase A consisted of H_2_O with 0.1% formic acid, and the mobile phase B consisted of methanol. The gradient flow was set at 1% (vol/vol) B for 7 min, linearly increased to 95% B in the next 0.1 min and maintained for 5 min, then linearly decreased to 1% B in 0.1 min, and finally maintained at this composition for an additional 7.8 min. The ESI source of TOF mass spectrometry detection was negative ion mode, spray voltage was 3 kV, and the capillary temperature was set to 300°C. The sheath gas and auxiliary gas were both nitrogens, the flow rates were 30 and 10 (arbitrary units), and the scan range set to 60 to 1,000 m/z. The pure 5-FU (CAS Number: 51–21-8) and FUPA (CAS Number: 5006–64-4) were purchased from Aladdin (Shanghai, China). The standard curves of 5-FU and FUPA were constructed by HPLC-based quantification of the peak area under a series concentration of 0.1 0.25, 0.5, 1, and 2 mM.

### Relative abundance calculation of *A. hadrus* and target genes

To investigate the relative abundance of *A. hadrus* and target genes, i.e., butyrate-producing genes and 5-FU metabolism genes, we downloaded the raw data of five cohorts from the Sequence Read Archive (SRA, https://www.ncbi.nlm.nih.gov/sra/), including a cohort of healthy males (41), an inflammatory bowel disease (IBD) cohort (42), a cohort of colorectal cancer (CRC) patients treated with FOLFOX (consisting of oxaliplatin and 5-FU) (43), a Chinese CRC cohort (44), and an Austrian CRC cohort (45). The metadata of five cohorts is detailed in Table S4. Then, we used Trim Galore (https://github.com/FelixKrueger/TrimGalore, version 0.6.7) and MultiQC (46) (version 1.13.dev0) to ensure the data quality. Finally, we applied Bowtie (47) (version 2.4.5) to remove potential host contamination (using the human genome sequence hg19 to build the index), resulting in clean data for subsequent analysis. All of the above software used default parameters. We calculated the relative abundance of *A. hadrus* and other species in five cohorts with MetaPhlAn (48) (version 3.0.14). Statistical analysis was performed using the ggpubr (49) package. Relative abundance on the species level was displayed using ggplot2 (50). We also used BWA (51) (version 0.7.17-r1188) to calculate the relative abundance of target genes with the following steps. First, butyrate-producing genes and 5-FU metabolism genes were extracted from the *A. hadrus* reference genome (GCF_000210695.1) to construct an index with BWA. Then, the BWA-MEM algorithm was chosen to align metagenomic data to the index. The number of mapped reads was calculated from the alignment results using Samtools (52) (version 1.6), and an R (53) (version 4.1.1) script was used to calculate TPM (transcripts per million) values. The specific calculation process of the R script is referenced from the study by Zhao et al. (54). Finally, TPM values were displayed using the pheatmap (55) package in R software.

## RESULTS

### Comparative genomic analysis uncovers an open pan-genome of *A. hadrus*

We screened and obtained 527 high-quality *A. hadrus* genomes from GenBank and UHGG, including 60 isolate genomes and 467 MAGs. The isolate genomes had an average size of 3.1 Mbp (range 2.8–3.4 Mbp), an average contig number of 114 (range 1–442), an average GC content of 37.0% (range 36.6%–37.5%), and an average N50 of 194.6 kbp (range 60.6 kbp–3.2 Mbp). The MAGs had an average size of 2.7 Mbp (range 2.5–3.3 Mbp), an average contig number of 168 (range 45–549), an average GC content of 37.2% (range 36.4%–38.1%), and an average N50 of 33.8 kbp (range 6.2–95.6 kbp). Each genome encoded an average of 2,555 predicted proteins (range 2,243–3,297).

We constructed the pan-genome of *A. hadrus* based on 527 genomes from 21 countries across four continents. The *A. hadrus* pan-genome contained 44,292 gene families, of which 1,196 were identified as core genes (present in more than 90% of 527 genomes), and the other 43,096 were identified as dispensable genes (present in less than 90% of 527 genomes). The average core gene content per *A. hadrus* genome was 46.8%. According to the Fig. S2A, the *A. hadrus* pan-genome is open, as the pan-genome size continuously increased with the addition of analyzed genomes. Moreover, the number of newly emerged gene families in the *A. hadrus* pan-genome decreased with the increase in analyzed genomes and eventually reached a plateau (Fig. S2B). When the number of analyzed genomes exceeds 500, adding each new genome resulted in an average of 46 new gene families. Thus, the *A. hadrus* pan-genome size expanded accordingly.

### Phylogenetic analysis reveals three clades in *A. hadrus* genomes

**Fig 1:**
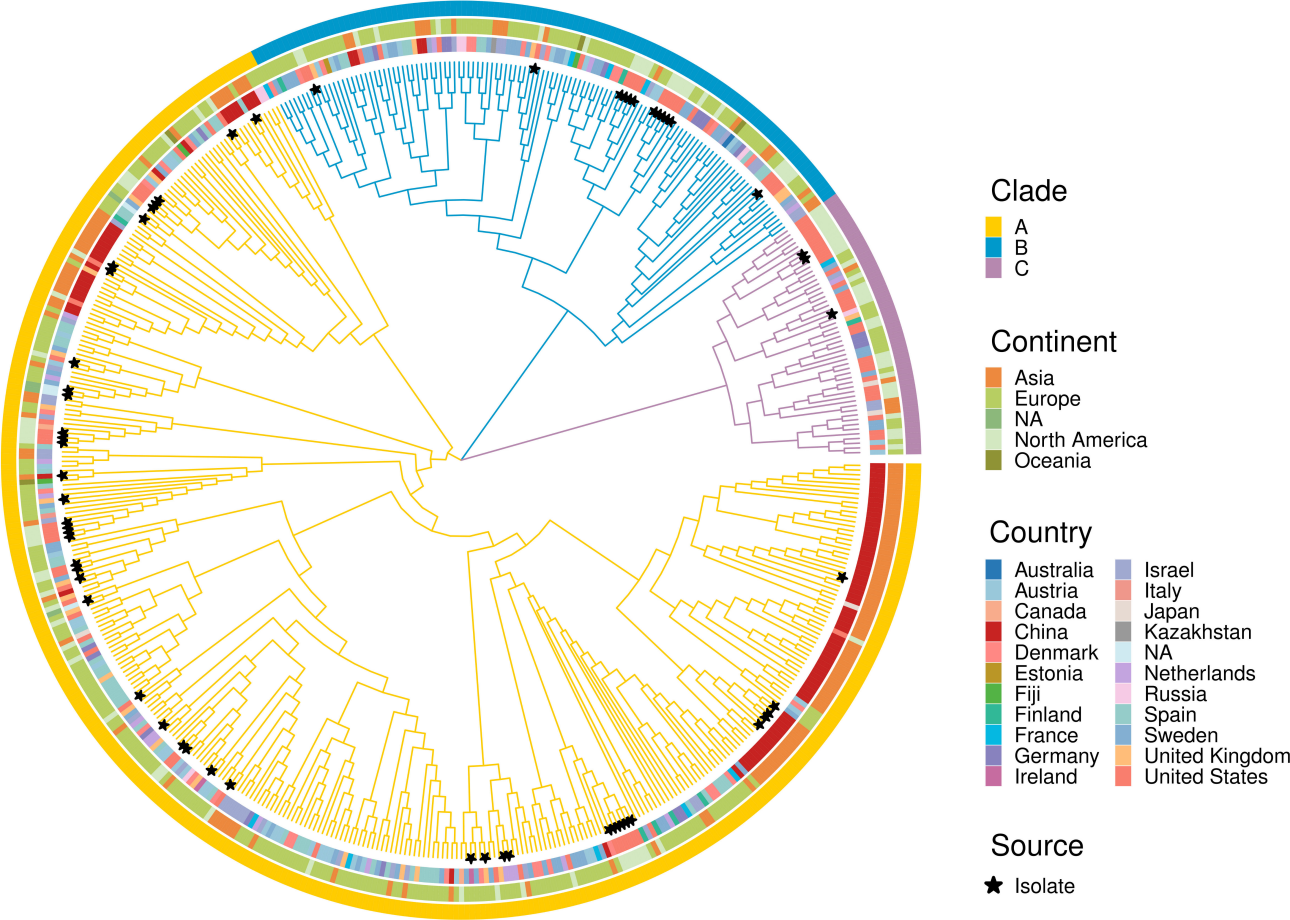
Phylogenetic tree of 527 *A*. *hadrus* genomes. The colors of the outer ring and branches represent different evolutionary clades. The middle and inner ring colors represent different continents and countries, respectively. The black stars represent isolate genomes.

To investigate the evolutionary relationships of *A. hadrus*, we generated a maximum likelihood phylogenetic tree using 527 genomes. We observed three distinct evolutionary clades, A, B, and C, as shown in Fig. 1. Clade A comprised 356 genomes, including 45 isolate genomes. Clades B and C contained 120 and 51 genomes, respectively, with 12 and 3 isolate genomes each. These clades were detected in Asia, Europe, and North America, except clade C, which was not detected in Oceania. All three clades were observed in 10 countries, including the United States, Germany, France, Austria, and others. Clades A and B were found in China, Spain, Denmark, and Fiji. Clades A and C were both present in Japan. Clade A was detected only in Ireland, Italy, and Canada. Clade B was detected exclusively in Kazakhstan, Australia, and Estonia.

**Fig 2:**
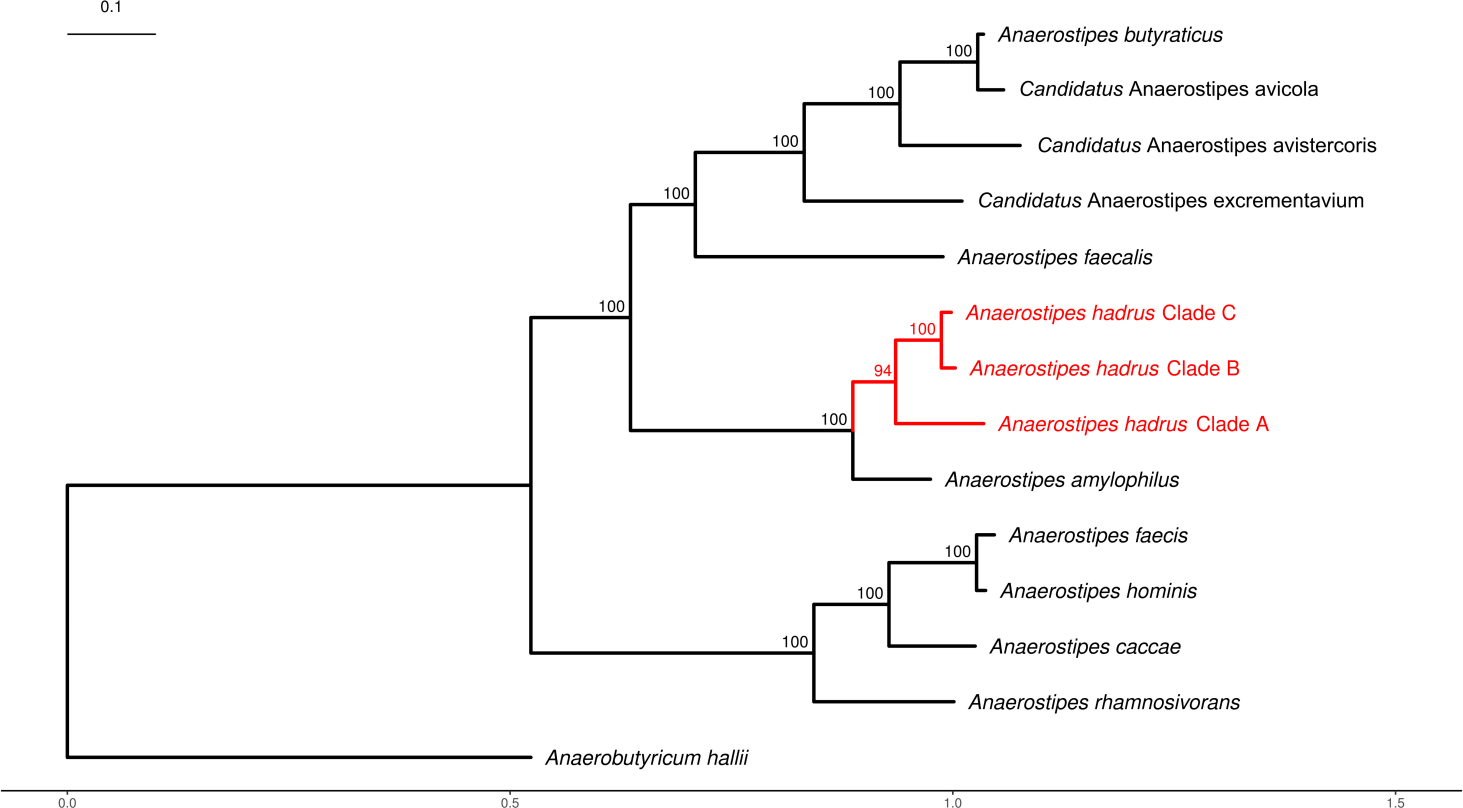
Phylogenetic tree of the genus *Anaerostipes*. This tree reflects the evolutionary relationships between *A. hadrus* and other species within the genus *Anaerostipes*. We designated *Anaerobutyricum hallii* as an outgroup. The scale bar represents 0.1 substitutions per nucleotide. Bootstrap values are presented as a percentage of 1,000 replications. Detailed accession numbers can be found in Table S3.

The genus *Anaerostipes* was first reported in 2002 (56). According to the NCBI Taxonomy database (http://www.ncbi.nlm.nih.gov/taxonomy) records, there are currently 11 species in the genus *Anaerostipes*, including eight confirmed and three candidatus species. We constructed a maximum likelihood phylogenetic tree of *Anaerostipes* species to study the evolutionary relationships between *A. hadrus* and other species (Fig. 2). The *Anaerostipes* species could be divided into two evolutionary clades. *A. faecis*, *A. hominis*, *A. caccae*, and *A. rhamnosivorans* existed on the smaller branch. While *A. butyraticus*, *A. faecalis*, *A. hadrus*, *A. amylophilus*, and three candidatus species were present on the bigger branch. *A. amylophilus* was the closest related species to *A. hadrus*. Compared to the minor clades B and C of *A. hadrus*, clade A was closer to *A. amylophilus*. The results of calculating the average nucleotide identity (ANI) between the three clades of *A. hadrus* also reflected this conclusion (Fig. S3). The ANI between 527 *A*. *hadrus* genomes was greater than 97%, and the average ANIs between clade A and clade B or clade C was 98.7%, while the mean ANI between clades B and C was 98.9%.

The three clades of *A. hadrus* differed in genome content. We used a Venn diagram to illustrate the dissimilarities in gene families (Fig. S4A). The most significant number of gene families, 13,652, was shared between clades A and B, while clades B and C shared the least number of gene families (8,949). Overall, there are 8,602 gene families shared among all three clades. Additionally, it was found that the larger the clade, the more specific genes it contained, with the most specific genes in clade A (*n* = 20,737) and the least specific genes in clade C (*n* = 2,677). Although the three clades of *A. hadrus* differed significantly in gene family members, functional annotation analysis suggested that the main functions of different clades were the same (Fig. S4B). Sorted by the number of annotated genes, the main KEGG pathways of the representative genomes from different clades involved ribosome, ABC transporters, pyruvate metabolism, glycolysis/gluconeogenesis, and purine metabolism.

### Widespread butyrate-producing genes drive the probiotic properties of *A. hadrus*

**Fig 3:**
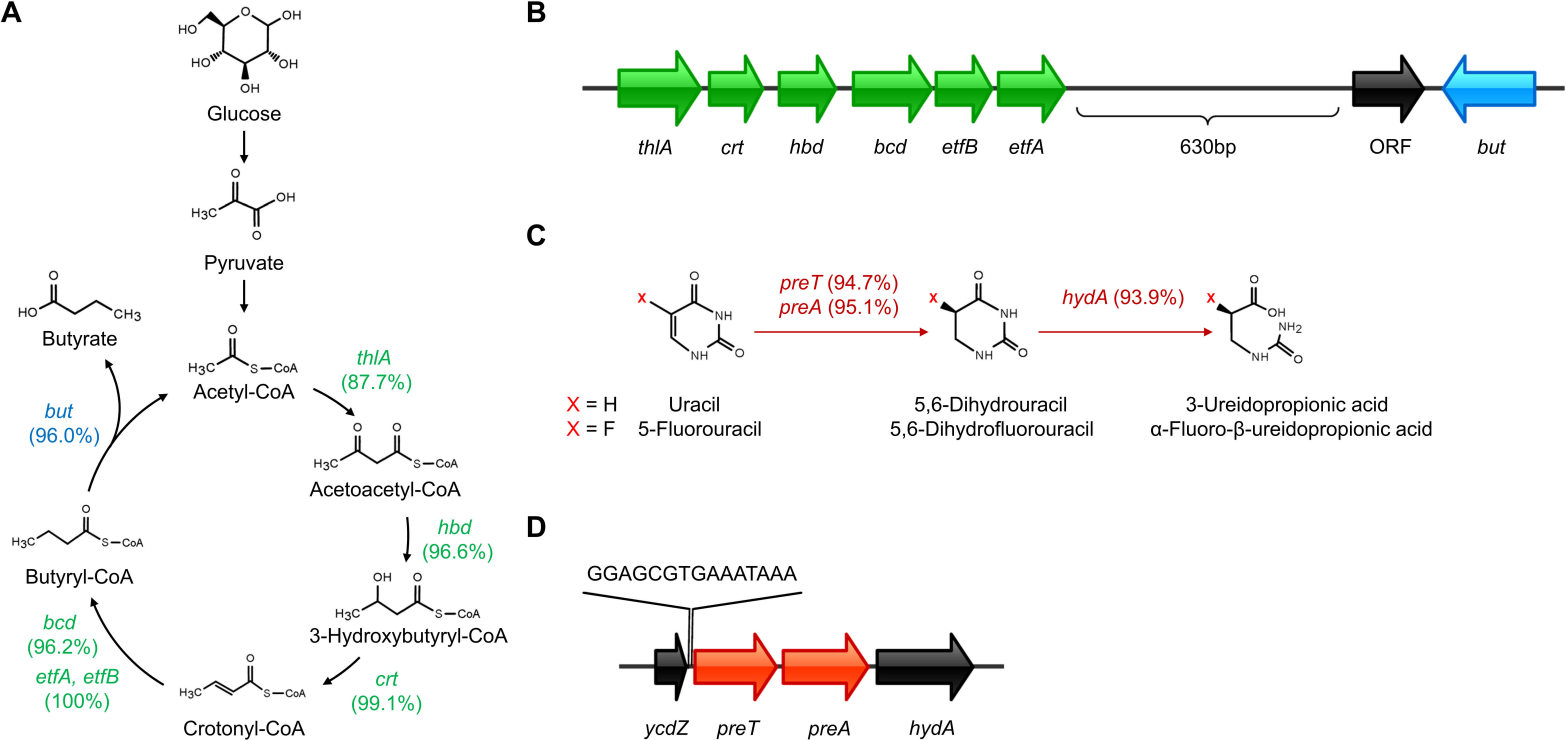
Target pathways and gene structures in *A. hadrus*. (**A)** Butyrate production pathway of *A. hadrus*. The numbers in parentheses indicate the gene frequency of 527 *A*. *hadrus* genomes. The genes and their encoded proteins are as follows: *bcd*, butyryl-CoA dehydrogenase; *but*, butyryl-CoA:acetate CoA-transferase; *crt*, short-chain-enoyl-CoA hydratase; *etfA*, electron transfer flavoprotein subunit alpha; *etfB*, electron transfer flavoprotein subunit beta; *hbd*, 3-hydroxybutyryl-CoA dehydrogenase; *thlA*, acetyl-CoA acetyltransferase. (**B)** The structure of butyrate-producing genes. Green genes are involved in the reduction of acetyl-CoA to butyryl-CoA. The blue gene participates in the last step of butyrate production. (**C)** Reductive pyrimidine catabolic pathway and 5-FU metabolism pathway of *A. hadrus*. The numbers indicate the gene frequency in 527 *A*. *hadrus* genomes. The genes and their encoded proteins are as follows: *hydA*, bacterial dihydropyrimidinase; *preA*, NAD-dependent dihydropyrimidine dehydrogenase subunit PreA; *preT*, NAD-dependent dihydropyrimidine dehydrogenase subunit PreT. (**D)** The structure of 5-FU metabolism genes. The gene structure was displayed using IBS (60) software.

Butyrate is produced by the condensation of two acetyl-CoA molecules (57). Seven genes in *A. hadrus* are involved in this process (58). We then calculated the frequency of all genes involved in the microbial synthesis of butyrate from *A. hadrus* genomes (Fig. 3A), with the lowest frequency of 87.7% (462/527) for *thlA* and the highest frequency of 100% (527/527) for *etfA* and *etfB*. There were 84.3% (444/527) genomes carrying all seven butyrate-producing genes. In general, butyrate-producing genes were arranged in the order of catalyzed reactions in *A. hadrus* genomes, except for *crt* and *hbd* (Fig. 3B). The first six genes were arranged in the same direction and are less spaced apart (<100 bp). In contrast, *but* was farther from the first six genes (>1,600 bp). Between *etfA* and *but*, there existed an open reading frame (ORF) with an unknown function (>1,000 bp), which was aligned in the opposite direction to *but*. Additionally, we identified potentially harmful genes from the Comprehensive Antibiotic Resistance Database [CARD (40)] and the Virulence Factor Database [VFDB (59)] in *A. hadrus* genomes. Only 12.3% (65/527) of the *A. hadrus* genomes carried one to five antibiotic resistance genes (mainly related to antibiotics such as aminoglycosides, tetracyclines, and lincosamides). Concurrently, no virulence factors related to biological processes of invasion and exotoxin were identified in 527 *A*. *hadrus* genomes. To validate the probiotic properties of *A. hadrus*, we determined the production of butyrate and the other commonly found SCFAs by *in vitro* assays. The results revealed that the *A. hadrus* CGMCC 1.32965 were able to produce linear chain SCFAs as acetic, propanoic, butyric, and valeric acids during *in vitro* fermentation in modified mGAM media, other than branch chain ones represented by isobutyric and isovaleric acids (Fig. S5). The yields of C2–C4 SCFAs were 2.99, 22.16, 137.95, and 3.32 mg/L, respectively.

### Ubiquitous 5-FU metabolism genes imply the complex role of *A. hadrus*

The *preTA* operon was first identified in *Escherichia coli* (*E. coli*) and encoded the bacterial dihydropyrimidine dehydrogenase (EcDPD) (61). EcDPD is not only involved in *E. coli* pyrimidine metabolism but also metabolizes 5-FU to the inactive dihydrofluorouracil (DHFU), functioning as human dihydropyrimidine dehydrogenase (DPD) (18). We identified the *preTA* operon in *A. hadrus*, suggesting that *A. hadrus* may have a similar reductive pyrimidine catabolic pathway as *E. coli* (Fig. 3C). In *A. hadrus* genomes, *preT* is located upstream of *preA*, and the two were adjacent to each other to form the *preTA* operon (Fig. 3D). *ycdZ*, the upstream gene of *preT* with unknown function, encoded a DUF1097 domain-containing protein with 38.4% amino acid identity to the intracellular membrane protein encoded by *E. coli ycdZ*. The downstream gene of *preA*, *hydA*, can encode the bacterial dihydropyrimidinase. However, there was only a 38.2% amino acid identity between the dihydropyrimidinase derived from *A. hadrus* (AhDHP) and human dihydropyrimidinase (DHP). DHP was reported to metabolize the catalytic product of DPD (62). We found that 93.2% (491/527) of *A. hadrus* genomes carried *ycdZ*, *preTA* operon, and *hydA*. Between *ycdZ* and *preT*, a potential motif (15 bp) that may be involved in regulating the *preTA* operon was predicted (Fig. 3D). Additionally, we noticed that both *A. hadrus* and *E. coli* carried the *preTA* operon but with different frequencies. We analyzed 2,565 complete *E. coli* genomes in GenBank and found that only 57.7% (1,480) of the genomes carried *preT* and *preA*. In contrast, 93.9% (495) of *A. hadrus* genomes carried *preTA* operon. Among 527 *A*. *hadrus* genomes analyzed in this study, the number of genomes carrying seven butyrate-producing genes and the *preTA* operon is 79.1% (417/527). This proportion reached 95% (57/60) in *A. hadrus* isolate genomes, indicating that the *preTA* operon metabolizing 5-FU was widely distributed within *A. hadrus* genomes as butyrate-producing genes.

**Fig 4:**
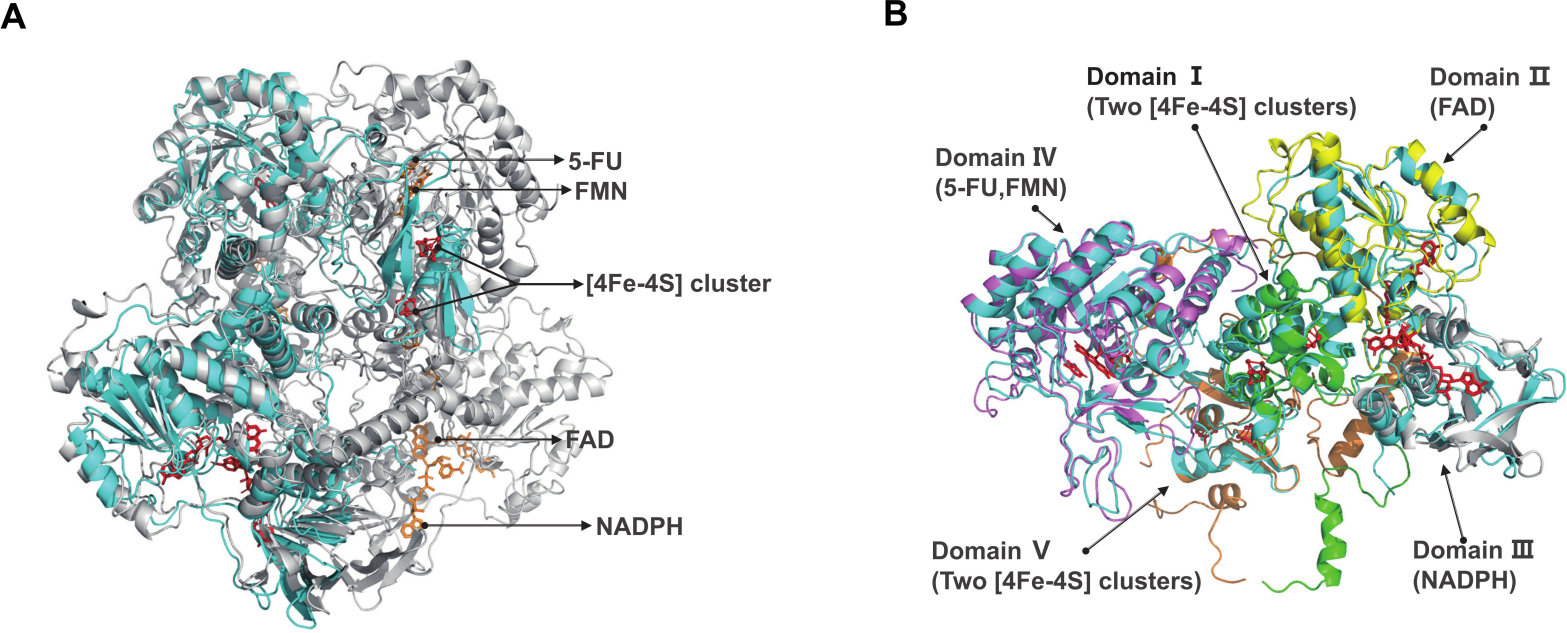
Structural basis of 5-FU metabolism in *A. hadrus*. (**A)** Structure comparison between the predicted *A. hadrus* PreT-PreA heterodimer (cyan) and the pig DPD (PDB ID: 1h7x) (gray). Co-factors and substrates on the same monomer of the pig DPD are represented by the same color (red or orange). (**B)** Structure comparison between the predicted *A. hadrus* PreT-PreA heterodimer (cyan) and five distinct domains of the pig DPD (PDB ID: 1h7x) monomer. Domains are represented by green, yellow, gray, purple, and orange, respectively.

Mammalian DPD is a homodimer, whereas EcDPD is a heterotetramer consisting of two PreT and two PreA subunits (63). Moreover, the *E. coli* PreT-PreA heterodimer function is similar to one pig DPD monomer (63). Sequence alignment showed 58% amino acid identity between *preT* genes and 65% amino acid identity between *preA* genes encoded by *A. hadrus* and *E. coli*. Thus, the *A. hadrus* PreT-PreA heterodimer may also have similar functions with the pig DPD monomer. To demonstrate this, we used ColabFold to predict a high-quality protein structure of *A. hadrus* PreT-PreA heterodimer (predicted LDDT score = 94.6, predicted TM score = 0.915). Through comparing with the crystal structure of a ternary complex consisting of pig DPD, NADPH, and 5-FU (PDB ID: 1h7x), we found that the *A. hadrus* PreT-PreA heterodimer (820 AA) had a similar structure [root-mean-square deviation (RMSD) = 1.703 Å for 624 Cα atoms] to the pig DPD monomer (1,025 AA) (Fig. 4A). There are five functionally distinct domains (domains I–V) in the pig DPD monomer (11179210). We further performed a structure alignment between these five domains and predicted structure for a more in-depth study of the *A. hadrus* PreT-PreA heterodimer function (Fig. 4B). We found the similar domain I (RMSD = 0.786 Å for 85 Cα atoms), domain II (RMSD = 0.596 Å for 116 Cα atoms), domain III (RMSD = 2.284 Å for 85 Cα atoms), domain IV (RMSD = 0.619 Å for 255 Cα atoms), and domain V (RMSD = 1.023 Å for 79 Cα atoms) at corresponding positions of the predicted structure. In addition, structure-based amino acid sequence alignment showed that almost all sites involved in binding Fe–S clusters, FAD, NADPH, 5-FU, and FMN within pig DPD monomer were matched in the PreT-PreA heterodimer protein sequence of *A. hadrus* (Table S5). Therefore, *A. hadrus* DPD (AhDPD) encoded by the *preTA* operon is theoretically a heterotetramer, which has the same potential to metabolize 5-FU as EcDPD.

**Fig 5:**
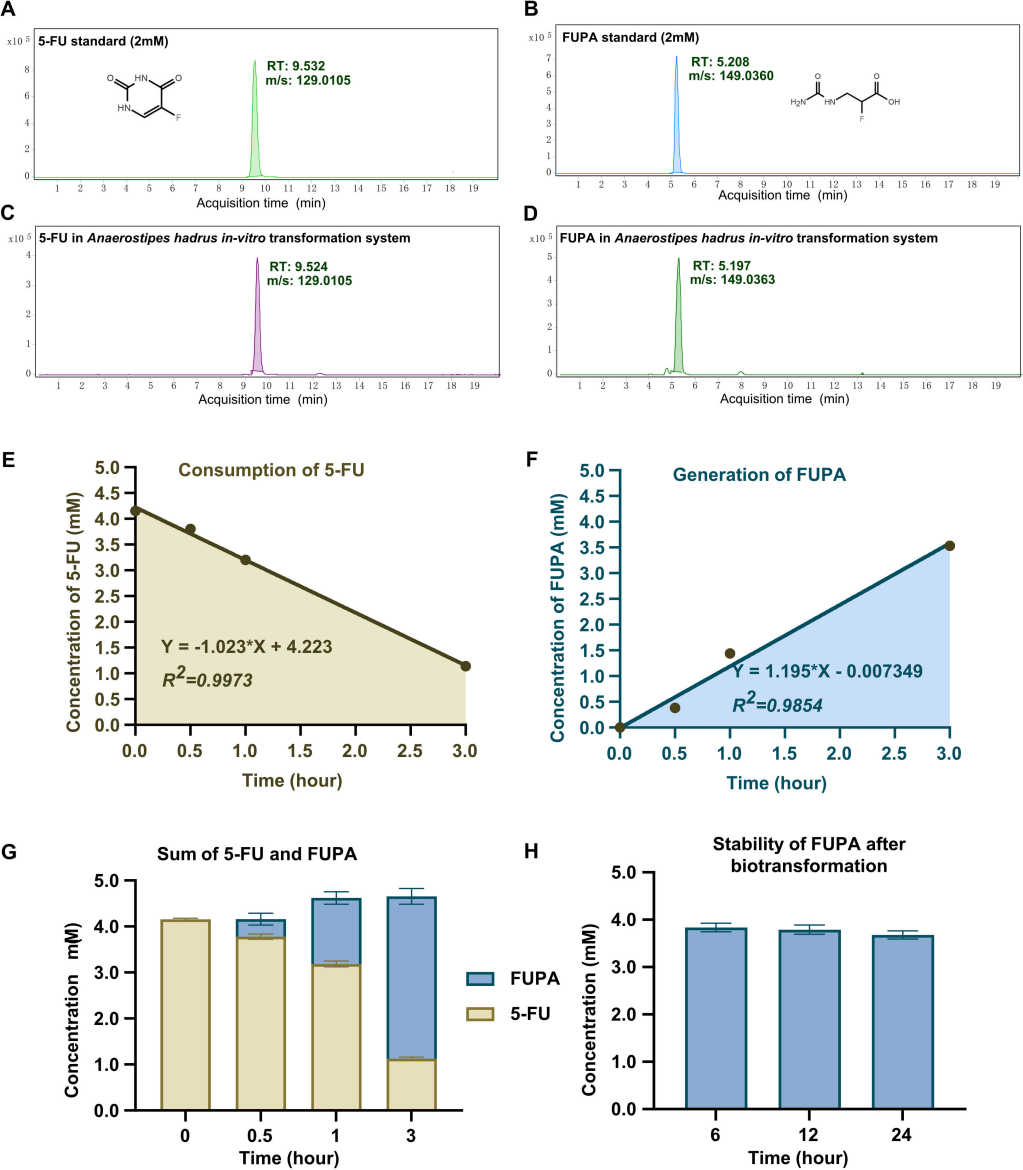
*A. hadrus*-mediated biotransformation of α-fluoro-β-alanine into α-fluoro-β-ureidopropionic acid *in vitro*.** (A–D)** The extracted iron chromographs of 5-FU standard (**A**), FUPA standard (**B**), and the remaining 5-FU (**C**) and generated FUPA (**D**) after 1 h of biotransformation by *A. hadrus*; RT, retention time; m/z, mass-to-charge ratio under negative source (−H). (**E–F)** The consumption of 5-FU (**E**) and generation of FUPA (**F**) by *A. hadrus*. The equation shown in the panel was calculated by simple linear regression analyzed by GraphPad Prism 9.0.** (G)** The total molar concentration of 5-FU and FUPA in one system after biotransformation by *A. hadrus* at different times.

Considering the presence of *hydA* at the downstream of *preTA* operon, which encodes the bacterial dihydropyrimidinase, we deduced that the *A. hadrus* was able to transform 5-FU into α-fluoro-β-ureidopropionic acid (FUPA) as dead-end product other than DHFU as previously reported (18). Such deduction was then verified by *in vitro* biotransformation assay as described in the Materials and Methods section. We observed that the 5-FU was consumed in the presence of *A. hadrus* CGMCC 1.32965 in the system followed by the gradual generation of FUPA (Fig. 5A through G). Further regression analysis revealed that every 10^9^ cells of *A. hadrus* CGMCC 1.32965 transform 5-FU into FUPA at an average velocity of 2.43 ± 1.66 mM/h. The level of the generated FUPA remained stable after an 18-h additional biotransformation (Fig. 5H), which indicated that the FUPA was a dead-end product that could not be transformed any further by *A. hadrus* CGMCC 1.32965.

### High prevalence of *A. hadrus* and preTA orthologues across diverse cohorts

**Fig 6:**
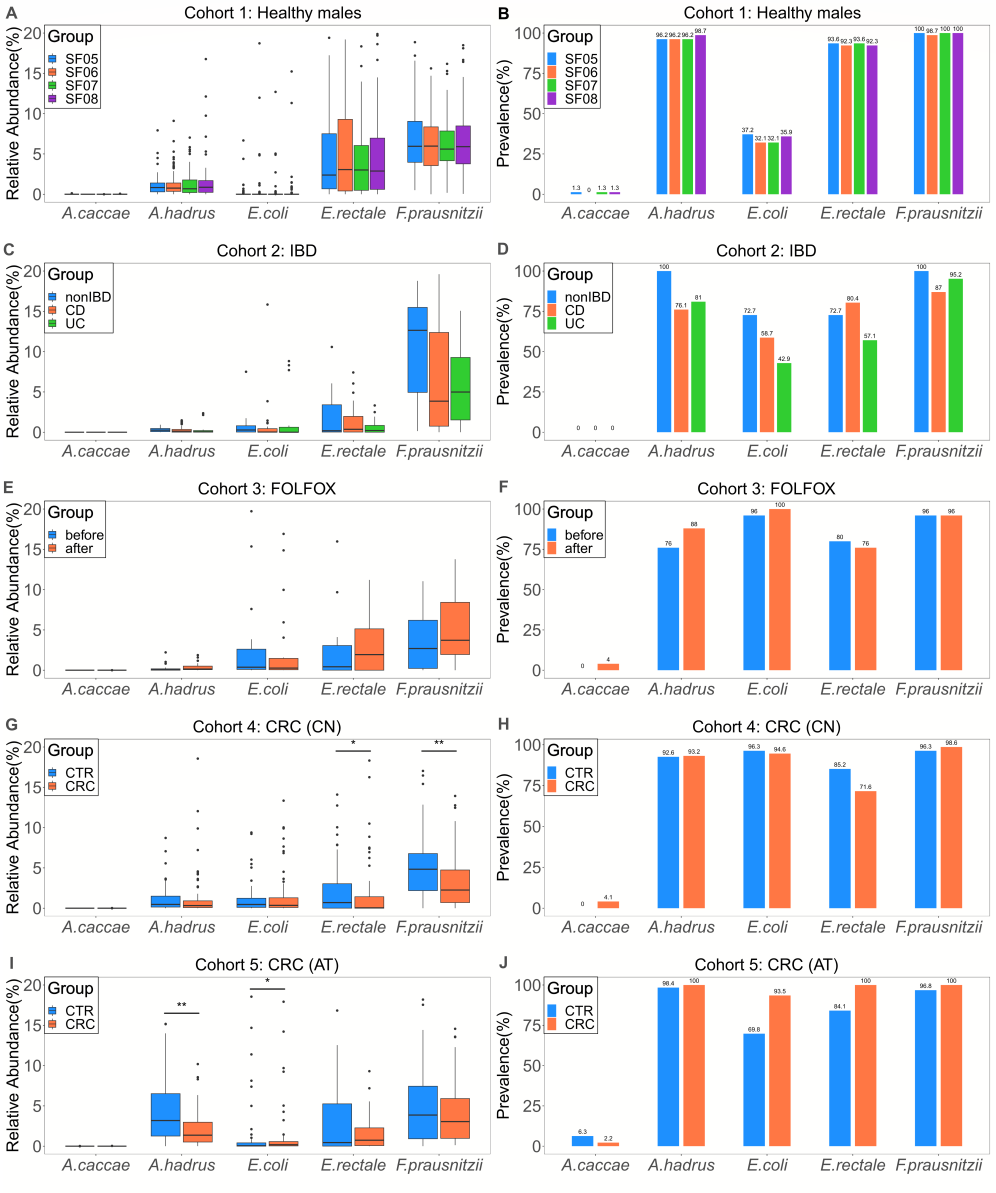
Relative abundance and prevalence of *A. hadrus* and other species. The relative abundance is presented in the left boxplots, while the prevalence is shown in the right barplots. (**A, B)** Cohort 1 comprises 78 healthy males who contributed four stool samples over 6 months, resulting in 312 metagenomic data sets. (**C, D** Cohort 2 includes 28 individuals, comprising 15 with CD, nine with UC, and four non-IBD controls, who provided multiple stool samples over a year, resulting in 78 metagenomic data sets. (**E, F)** Cohort 3 includes 25 CRC patients who provided one stool sample before and after taking FOLFOX, resulting in 50 metagenomic data sets. (**G, H)** Cohort 4 consists of 128 Chinese individuals, including 74 with CRC and 54 healthy controls, who provided one stool sample each, resulting in 128 metagenomic data sets. (**I, J)** Cohort 5 comprises 109 Austrian individuals, 46 with CRC and 63 healthy controls, who provided one stool sample each, resulting in 109 metagenomic data sets. Statistical analysis was performed by a Wilcoxon rank sum test (*P < 0.05, **P < 0.01).

To further investigate the distribution of *A. hadrus*, we calculated its relative abundance in five cohorts. To better illustrate the relative abundance of *A. hadrus*, we compared *A. hadrus* with four other bacteria (Fig. 6), including *Anaerostipes caccae* (*A. caccae*), *E. coli*, *Escherichia rectale* (*E. rectale*), and *Faecalibacterium prausnitzii* (*F. prausnitzii*)*. A. caccae* is the type species of the genus *Anaerostipes* and can produce butyrate (56). *E. coli* is a common conditional pathogen in the human intestinal tract and carries the *preTA* operon as *A. hadrus. E. rectale* and *F. prausnitzii* are high-abundance butyrate-producing bacteria in the human colon with essential healthy effects (10). In cohort 1, we found that the relative abundance and prevalence of *A. hadrus* in healthy men remained stable at different time points and were higher than that of *E. coli* (Fig. 6A and B). In cohort 2, compared with non-IBD individuals, the prevalence of *A. hadrus* in IBD patients decreased to 76.1%, still higher than that of *E. coli* (Fig. 6D), but its relative abundance did not change significantly (Fig. 6C). In cohort 3, after receiving FOLFOX treatment, *A. hadrus* prevalence in CRC patients increased (Fig. 6F). In cohorts 4 and 5, we found that almost all samples from CRC patients and healthy individuals carried *A. hadrus* (Fig. 6H and J). In these five metagenomic cohorts, only *A. hadrus* and *A. caccae* were detected within the genus *Anaerostipes*, with the relative abundance and prevalence of *A. hadrus* being much higher than that of *A. caccae*. Overall, in most healthy individuals, IBD patients, and CRC patients, the relative abundance of *A. hadrus* remained stable at less than 5%. Nevertheless, in some healthy individuals and CRC patients, the relative abundance of *A. hadrus* could reach around 15%. In addition, the prevalence rate of *A. hadrus* is similar to that of *E. rectale* and *F. prausnitzii*, maintaining a high level.

**Fig 7:**
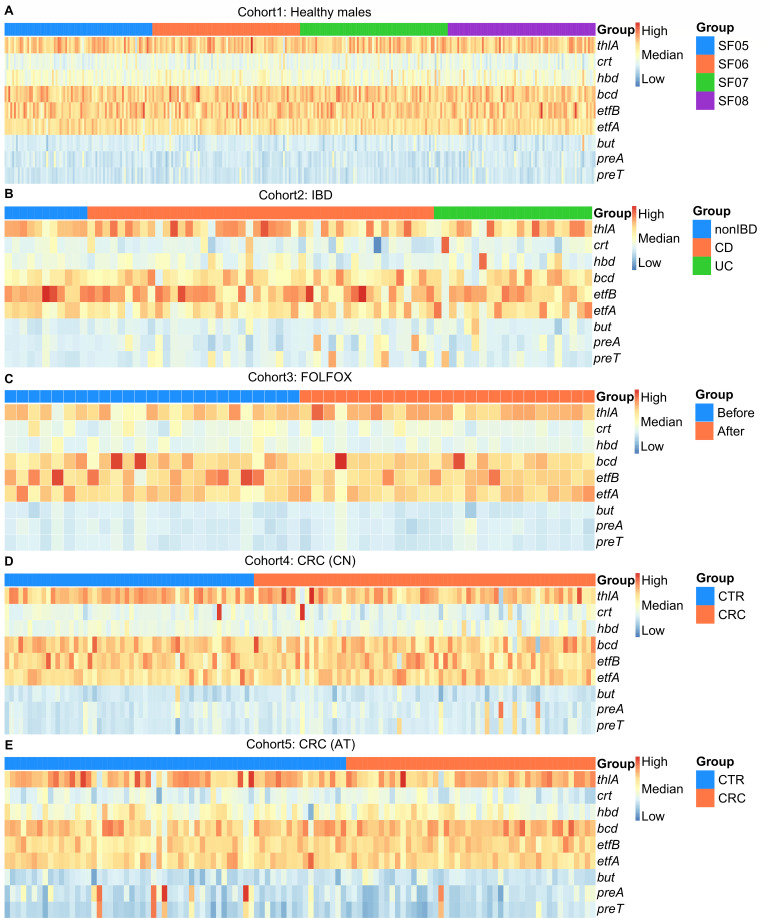
Relative abundance of butyrate-producing genes and 5-FU metabolism genes. The warmer color indicates a higher TPM value of the gene, corresponding to a higher relative abundance in one sample.

We further investigated the relative abundance of nine target genes, including butyrate-producing genes (*thlA*, *crt*, *hbd*, *bcd*, *etfB*, *etfA*, and *but*) and 5-FU metabolism genes (*preT* and *preA*), in five metagenomic cohorts. We found that these nine genes remained stable in relative abundance across cohorts and did not differ clearly between subgroups of the same cohort (Fig. 7). Overall, the relative abundance of *thlA*, *bcd*, etfB, and etfA was similar and at a higher level, while the relative abundance of *but*, *preT*, and *preA* was similar but lower. Additionally, in cohorts 4 and 5, we found that the relative abundance of *preT* and *preA* was significantly higher in a small number of samples from Chinese CRC patients, healthy Austrian individuals, and Austrian CRC patients.

## DISCUSSION

In this study, we conducted the first large-scale pan-genome analysis of *A. hadrus* (*n* = 527). We found that the proportion of core gene families in *A. hadrus* pan-genome (2.7%) is slightly lower than in other species (3%–84%) (64). Compared with other butyrate-producing bacteria, including *F. prausnitzii* [4.5% of core gene families in the pan-genome constructed by 84 strains (65)], *Clostridium perfringens* [3.8% of core gene families in the pan-genome constructed by 173 strains (66)], and *Clostridium butyricum* [9.9% of core gene families in the pan-genome constructed by 32 strains (67)], *A. hadrus* had a smaller core genome, suggesting the functional diversity and complexity. The phylogenetic and functional annotation analysis results showed no noticeable geographical distribution differences among different *A. hadrus* clades (Fig. 1), and their main functions were broadly consistent (Fig. S4B). However, we still need to pay attention to the impact of geographic factors on *A. hadrus* genomes. The latest study pointed out that the *A. hadrus* genome was prone to structural variations, and the core gene sequence identity cannot fully reflect functional similarity among *A. hadrus* genomes (68). Since a higher proportion of dispensable genes is in the *A. hadrus* pan-genome, the influence of strain isolation environment on dispensable genes should be fully considered when studying the function of a single *A. hadrus* strain.

5-FU is a first-line drug for chemotherapy in patients with CRC. However, host-derived DPD, DHP, and β-ureidopropionase from the reductive pyrimidine catabolic pathway (62) successively metabolize the majority of 5-FU entering the human body into non-anticancer DHFU, FUPA, and α-fluoro-β-alanine (69). In this study, we demonstrated that the *A. hadrus* genomes harbored homologs of human DPD and DHP encoded by the *preTA* operon and *hydA*, and observed for the first time that *A. hadrus* can convert 5-FU to FUPA. Concurrently, no homolog of β-ureidopropionase was found in any of the 527 *A*. *hadrus* genomes, which explains that the final product of 5-FU metabolism by *A. hadrus* is FUPA other than α-fluoro-β-alanine. Furthermore, we found that the location of *hydA* in *E. coli* genomes is far from the *preTA* operon, which may be the reason *E. coli* metabolizes 5-FU into DHFU (18), indicating that the conservation of the reductive pyrimidine catabolic pathway varies among different bacteria. Since humans and various microorganisms metabolize 5-FU into different final products, this character may help us distinguish different participants in 5-FU metabolism.

Although *A. hadrus* may interfere with the therapeutic effect of 5-FU due to the presence of the *preTA* operon, on the other hand, *A. hadrus* is expected to become probiotics for CRC patients suffering from DPD deficiency. It has been reported that 10%–30% of patients experience severe adverse reactions after receiving fluoropyrimidine treatment, and 30%–80% of them are due to the lack of DPD (70). In theory, *A. hadrus* can exert the same 5-FU rate-limiting effect as mammalian DPD and produce beneficial butyrate for the human body. Thus, *A. hadrus* has broad application prospects in helping CRC patients reduce 5-FU toxicity. Through metagenomic analysis, this study revealed that the distribution of *A. hadrus* was characterized by wide and stable features across different cohorts (Fig. 6). Notably, in the cohort consisting of non-IBD individuals and IBD patients, the prevalence of *A. hadrus* in stool samples from CD and UC patients was significantly lower, suggesting that butyrate-producing *A. hadrus* may be associated with the occurrence and development of IBD. Besides, in the cohort of CRC patients treated with FOLFOX, we found that this first-line chemotherapeutic agent increased the prevalence of *A. hadrus* in CRC patients' stool samples. Previous studies have indicated that the gut microbiome regulates the efficacy of FOLFOX (71, 72). Thus, it is worth exploring whether the increase in *A. hadrus* abundance will affect the subsequent therapeutic effect of FOLFOX. Our study also found that the relative abundance of *preT* and *preA* was close to that of *but*. In nature, most butyrate-producing bacteria rely on the butyryl-CoA:acetate CoA transferase encoded by *but* to complete the final step of butyrate production (73). Therefore, we should take seriously the potential impact of *preT* and *preA* from gut microbiota on fluoropyrimidine drugs. Additionally, we found that samples with a higher abundance of *A. hadrus* carried more *preT* and *preA*, which may support the idea that *preTA* operons in the population are mainly derived from *Anaerostipes* (18).

Despite exploratory analyses, there are still some limitations in our study. First, due to the strict culture conditions, the genome resources of *A. hadrus* strains that can be publicly obtained for analysis are limited. Therefore, we incorporated more MAGs to study the *A. hadrus* pan-genome comprehensively. However, MAGs can cause the loss of core genes (74), so the core genome size of the *A. hadrus* pan-genome we described may be slightly smaller than the actual situation. Second, although we calculated the relative abundance of butyrate-producing genes and 5-FU metabolism genes in five metagenomic cohorts, this only preliminary indicated that different populations carry a certain number of *preT* and *preA*. More research is needed on the level of gene expression. Third, our functional description of *A. hadrus* needs to be entirely adequate. Through protein structure prediction and amino acid sequence alignment, we speculated the binding sites of co-factors and substrates in AhDPD (Table S8). Nevertheless, these speculations have yet to be verified due to experimental limitations.

### Conclusion

Through a large-scale *A. hadrus* population analysis, we systematically studied the evolutionary relationship of *A. hadrus* and found that butyrate-producing genes and genes involved in 5-FU metabolism (the *preTA* operon and *hydA*) are core genes. Through culture-based *in vitro* biotransformation assay, we then confirmed that the *A. hadrus* metabolizes 5-FU into FUPA as dead-end product for the first time. Based on the distribution of *A. hadrus*, *preT*, and *preA* in different metagenomic cohorts, we suggested that butyrate-producing *A. hadrus* may interfere with the efficacy of fluoropyrimidine drugs or reduce adverse reactions in CRC patients, which may depend on the level of human DPD. In conclusion, this study found that *A. hadrus* has the potential to exert beneficial or harmful effects on hosts, which expands our understanding of bacterial duality and inspires us to study the role of *A. hadrus* in the human body deeply, to better apply *A. hadrus* to clinical diagnosis and treatment of related diseases.

## Data Availability

The genomic data of *A. hadrus* are available in Table S1. The raw sequencing data can be found in the National Center for Biotechnology Information Sequence Read Archive (https://www.ncbi.nlm.nih.gov/sra/) under the following identifiers: PRJNA354235, PRJNA389280, PRJNA484031, PRJEB10878, PRJEB7774. Metadata for these samples are available in Table S6.

## SUPPLEMENTAL MATERIAL

Supplemental figures msphere.00816-23-s0001.pdf Figures S1 to S5.

Legends msphere.00816-23-s0002.docx Supplemental material legends.

Table S1 msphere.00816-23-s0003.xlsx Basic information of 527 publicly available *A. hadrus* genomes.

Table S2 msphere.00816-23-s0004.xlsx Quality assessment results of 527 *A. hadrus* genomes.

Table S3 msphere.00816-23-s0005.xlsx Basic information of representative genomes for constructing the phylogenetic tree of the genus *Anaerostipes*.

Table S4 msphere.00816-23-s0006.xlsx Basic information of the five cohorts used in this study.

Table S5 msphere.00816-23-s0007.pdf Structure-based amino acid sequence alignment between predicted *A. hadrus* PreT-PreA heterodimer and five pig DPD domains.

ASM does not own the copyrights to Supplemental Material that may be linked to, or accessed through, an article. The authors have granted ASM a non-exclusive, world-wide license to publish the Supplemental Material files. Please contact the corresponding author directly for reuse.
