# Supplementary material for: Anaerostipes hadrus, a butyrate-producing bacterium capable of metabolizing 5-fluorouracil
Source: mSphere. 2024 Mar 12;9(4):e00816-23. doi: 10.1128/msphere.00816-23 (PMC11036815; doi:10.1128/msphere.00816-23)
Supplement: Supplemental figures — Figures S1 to S5. [file msphere.00816-23-s0001.pdf]

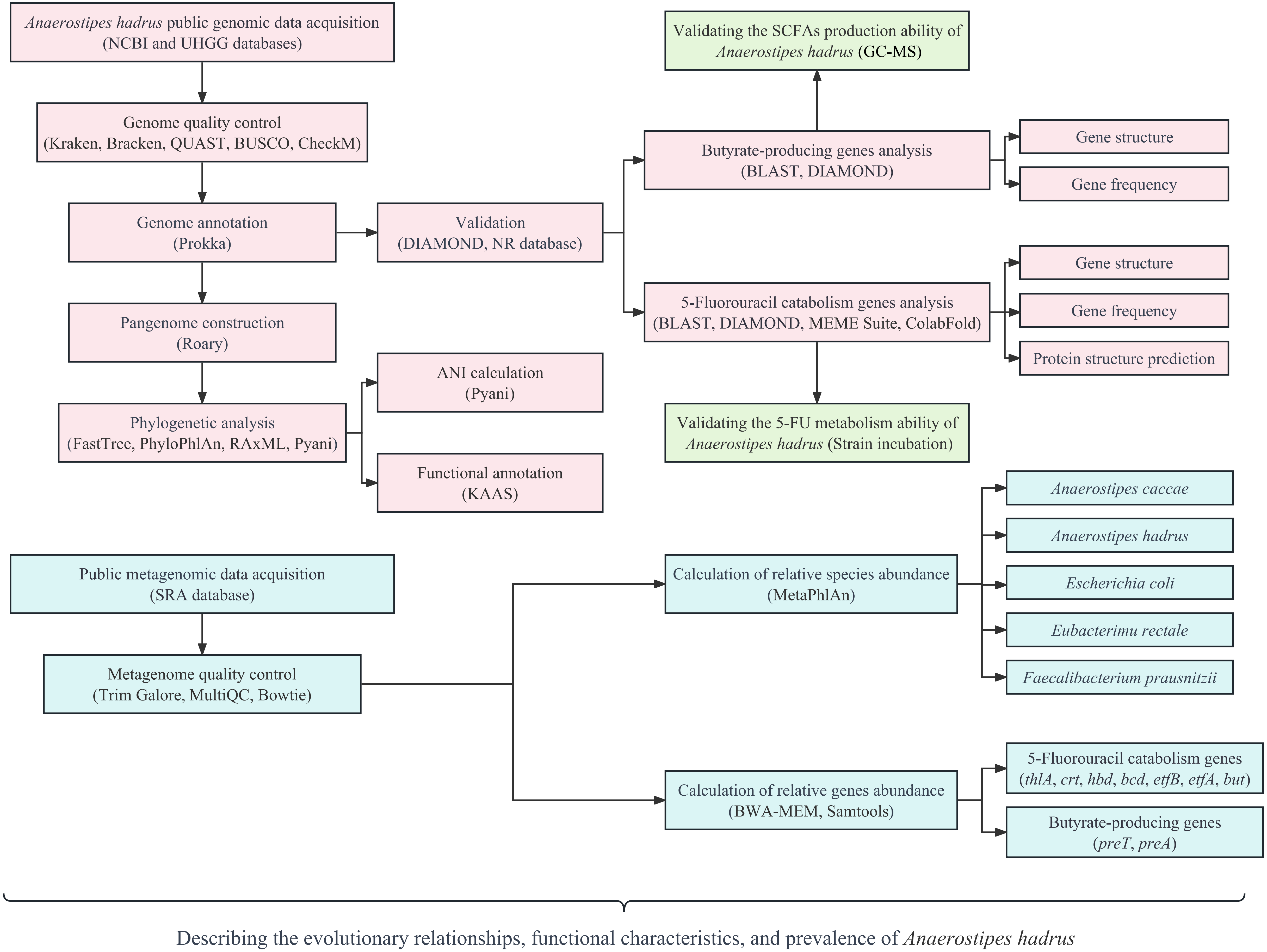
Anaerostipes hadrus public genomic data acquisition (NCBI and UHGG databases)
Genome quality control (Kraken, Bracken, QUAST, BUSCO, CheckM)
Genome annotation (Prokka)
Validation (DIAMOND, NR database)
Pangenome construction (Roary)
Phylogenetic analysis (FastTree, PhyloPhlAn, RAxML, Pyani)
ANI calculation (Pyani)
Functional annotation (KAAS)
Validating the SCFAs production ability of Anaerostipes hadrus (GC-MS)
Butyrate-producing genes analysis (BLAST, DIAMOND)
Gene structure
Gene frequency
5-Fluorouracil catabolism genes analysis (BLAST, DIAMOND, MEME Suite, ColabFold)
Gene structure
Gene frequency
Protein structure prediction
Validating the 5-FU metabolism ability of Anaerostipes hadrus (Strain incubation)
Public metagenomic data acquisition (SRA database)
Metagenome quality control (Trim Galore, MultiQC, Bowtie)
Calculation of relative species abundance (MetaPhlAn)
Anaerostipes caccae
Anaerostipes hadrus
Escherichia coli
Eubacterimu rectale
Faecalibacterium prausnitzii
Calculation of relative genes abundance (BWA-MEM, Samtools)
5-Fluorouracil catabolism genes (thlA, crt, hbd, bcd, etfB, etfA, but)
Butyrate-producing genes (preT, preA)
Describing the evolutionary relationships, functional characteristics, and prevalence of Anaerostipes hadrus

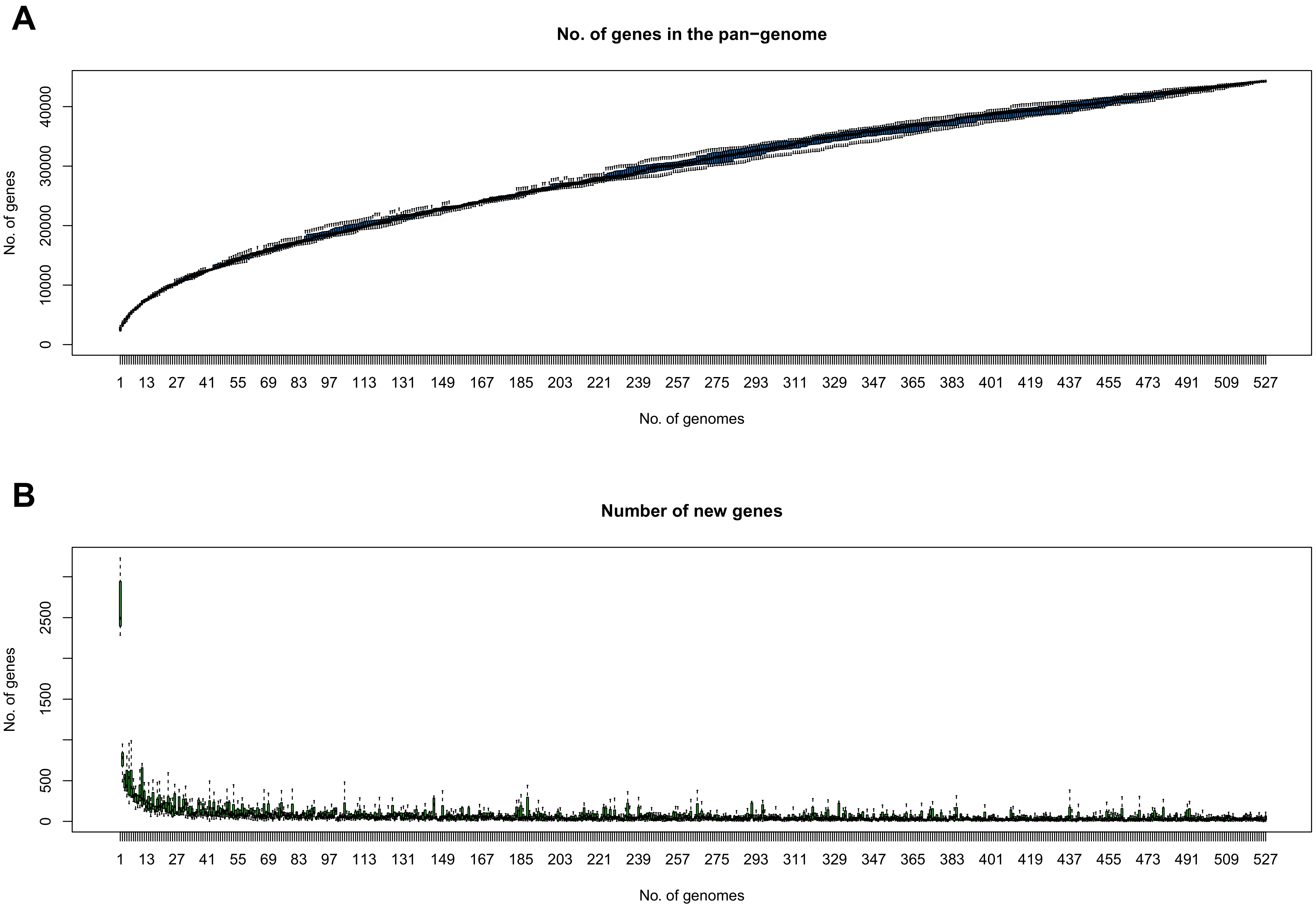
A
No. of genes in the pan−genome
No. of genes
0
10000
20000
30000
40000
1
13
27
41
55
69
83
97
113
131
149
167
185
203
221
239
257
275
293
311
329
347
365
383
401
419
437
455
473
491
509
527
No. of genomes
B
Number of new genes
No. of genes
0
500
1500
2500
1
13
27
41
55
69
83
97
113
131
149
167
185
203
221
239
257
275
293
311
329
347
365
383
401
419
437
455
473
491
509
527
No. of genomes

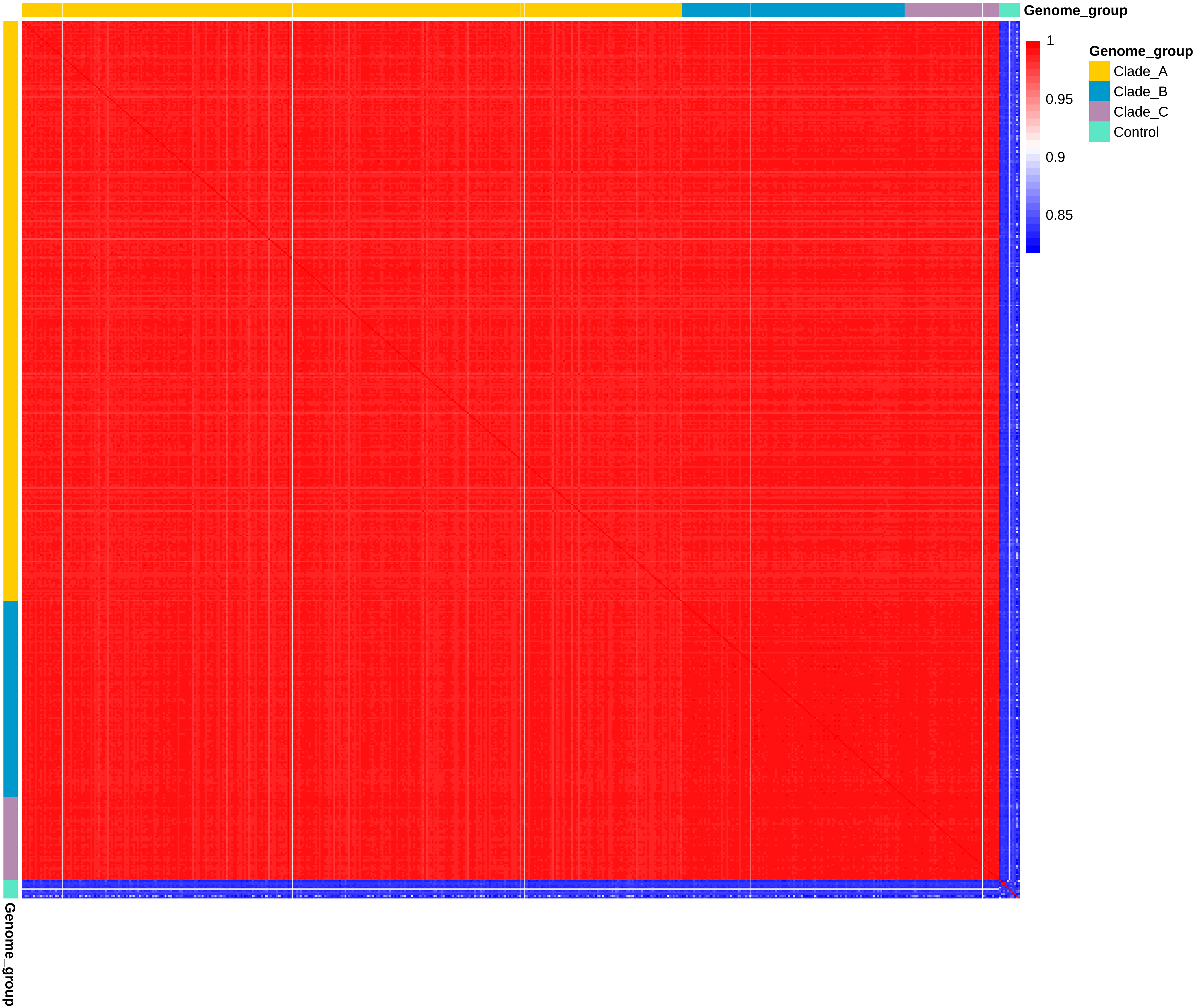
Genome_group
1
0.95
0.9
0.85
Genome_group
Clade_A
Clade_B
Clade_C
Control
Genome_group

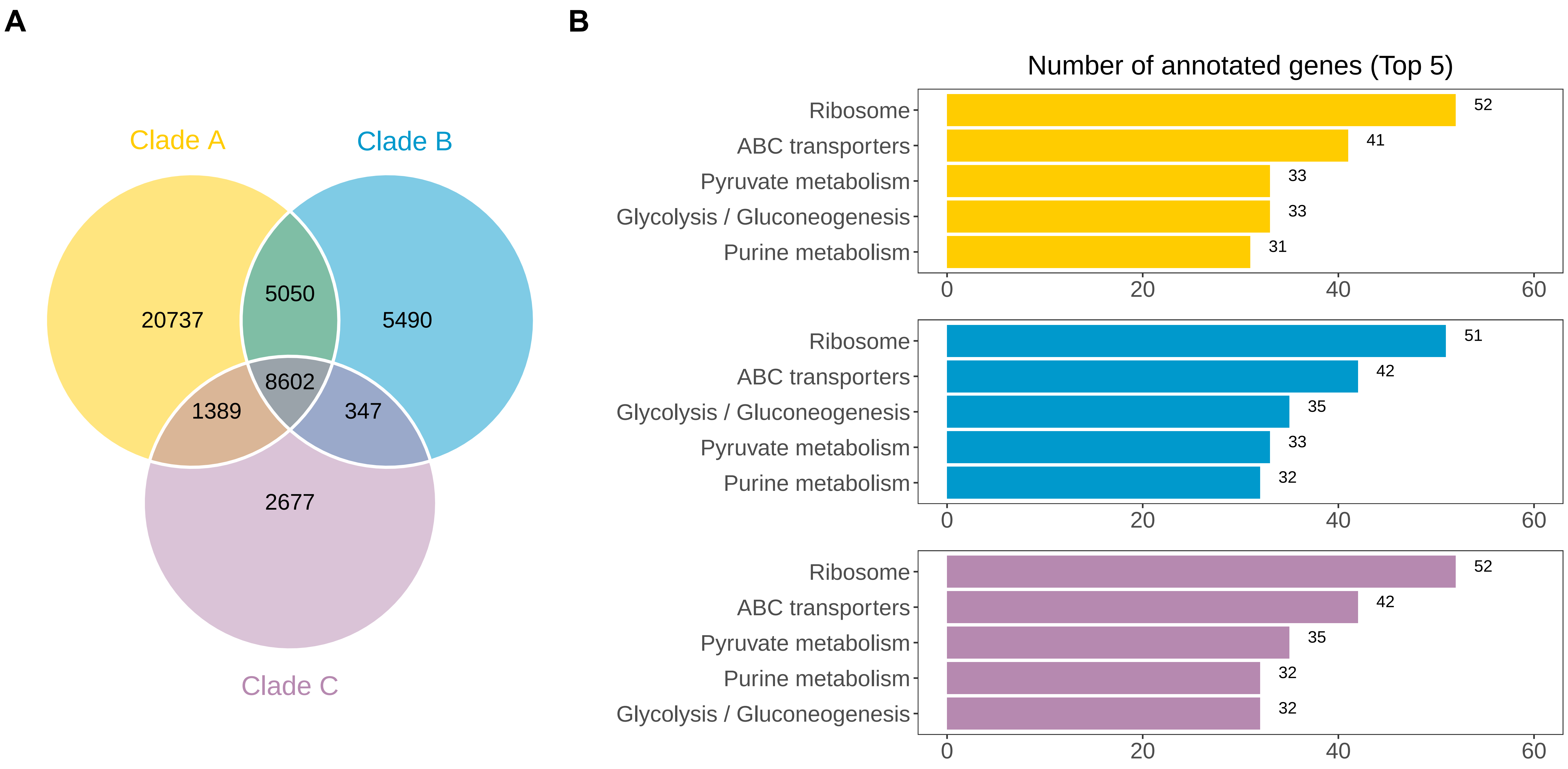
A
B
Clade A
Clade B
20737
5050
5490
8602
1389
347
2677
Clade C
Number of annotated genes (Top 5)
Ribosome
ABC transporters
Pyruvate metabolism
Glycolysis / Gluconeogenesis
Purine metabolism
52
41
33
33
31
Ribosome
ABC transporters
Glycolysis / Gluconeogenesis
Pyruvate metabolism
Purine metabolism
51
42
35
33
32
Ribosome
ABC transporters
Pyruvate metabolism
Purine metabolism
Glycolysis / Gluconeogenesis
52
42
35
32
32
0
20
40
60

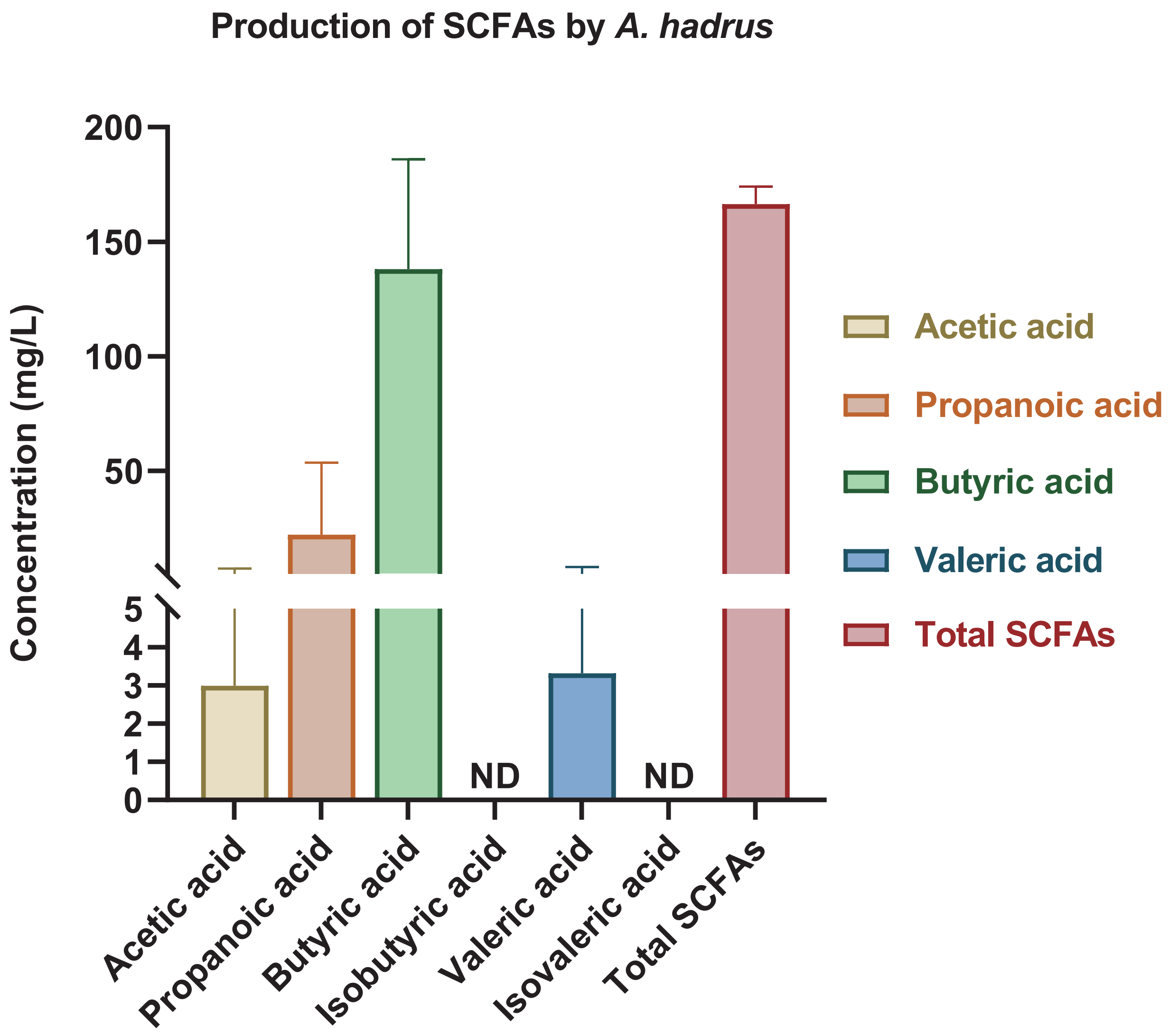
Production of SCFAs by A. hadrus
Concentration (mg/L)
200
150
100
50
5
4
3
2
1
0
ND
ND
Acetic acid
Propanoic acid
Butyric acid
Isobutyric acid
Valeric acid
Isovaleric acid
Total SCFAs
Acetic acid
Propanoic acid
Butyric acid
Valeric acid
Total SCFAs
