# Supplementary material for: Anaerostipes hadrus, a butyrate-producing bacterium capable of metabolizing 5-fluorouracil
Source: mSphere. 2024 Mar 12;9(4):e00816-23. doi: 10.1128/msphere.00816-23 (PMC11036815; doi:10.1128/msphere.00816-23)
Supplement: Legends — Supplemental material legends. [file msphere.00816-23-s0002.docx]

**Supplementary figure legends**

**Fig. S1.** Analysis workflow of this study.

**Fig. S2.** *A. hadrus* pan-genome analysis. (**A**), Variation of gene number with the genome size in *A. hadrus* pan-genome. (**B**), Variation of new gene number with the genome size in *A. hadrus* pan-genome.

**Fig. S3.** ANI heatmap of *A. hadrus* genomes. The control group contains species shown in Fig.2, except for *A. hadrus*.

**Fig. S4.** Differences between *A. hadrus* clades. (**A**), Venn diagram of gene number in three *A. hadrus* clades. (**B**), Barplot of functional annotation analysis on representative genomes from different *A. hadrus* clades.

**Fig. S5.** The production of short chain fatty acids (SCFAs) by *A. hadrus*.

**Supplementary table legends**

**Table S1.** Basic information of 527 publicly available *A. hadrus* genomes.

**Table S2.** Quality assessment results of 527 *A. hadrus* genomes.

**Table S3.** Basic information of representative genomes for constructing the phylogenetic tree of the genus *Anaerostipes*.

**Table S4.** Basic information of the five cohorts used in this study.

**Table S5.** Structure-based amino acid sequence alignment between predicted *A. hadrus* PreT-PreA heterodimer and five pig DPD domains. In the protein structure comparison, the predicted *A. hadrus* PreT-PreA heterodimer and its aligned sequences are represented in cyan. In contrast, the domains of the pig DPD and their aligned sequences are represented in gray. Amino acid sequences that do not align are represented in black. Sequences of other colors stand for the binding sites of different co-factors/substrates that are aligned. Underlines indicate binding sites that do not align.
