# Supplementary material for: Anaerostipes hadrus, a butyrate-producing bacterium capable of metabolizing 5-fluorouracil
Source: mSphere. 2024 Mar 12;9(4):e00816-23. doi: 10.1128/msphere.00816-23 (PMC11036815; doi:10.1128/msphere.00816-23)
Supplement: Table S5 — Structure-based amino acid sequence alignment between predicted A. hadrus PreT-PreA heterodimer and five pig DPD domains. [file msphere.00816-23-s0007.pdf]

Structure-based amino acid sequence alignment between predicted *A. hadrus* PreT-PreA heterodimer and five pig DPD domains

Alignment	Schematic Diagram	Result	Sequence
Domain Ⅰ (nFeS1,nFeS2)	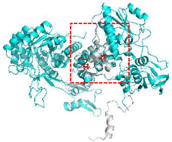	RMSD = 0.786 (85 to 85 atoms)	21 TAMEEASRCLLCHDAPCSKSCPAKTDPAKFIRSLRFRNIKGAAETIRENNPLAGCCAEVCPYGQLCEQACSRTGIDK-PIEI<br>GKLQKFLVEMEK 113<br>71 GALREAMRCLKCADAPCQKSCPTHLDIKSFITSISNKNYYGAAKMIFSDNPLGLTCGMVCPTSDLCVGGCNLYATEEGSINI<br>GGLQQFASEVFK 164
Domain Ⅱ (FAD)	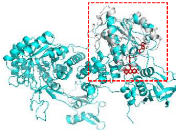	RMSD = 0.596 (116 to 116 atoms)	128 GKIACVGAGPASLACARELALFGY-DVTIYEANEKAGGVLTYGIVPSRLPQEVVDFDIATIEKLGVTFKFNETIDAARL--E<br>ELKAE-YDAVFVGTGL 221 369 VFTGGDMVNGGKTVVQAVAEGKDAAAMIAAYLN 401<br>188 AKIALLGAGPASISCASFLARLGYSDITIFEKQEYVGGLSTSEIPQFRLPYDVVNFEIELMKDLGVKIICGKSLSENEITLN<br>TLKEEGYKAAFIGIGL 285 476 VFAGGDIVGMANTTVESVNDGKQASWYIHKYIQ 508
Domain Ⅲ (NADPH)	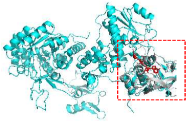	RMSD = 2.284 (85 to 85 atoms)	242 DFLK-DARLNKENM--------TLGDTVLVIGGGDVAMDCVTTAKQLGAKAT-IVYRR---TIEEAPADIDEVMAVQSMGIP<br>IIQEFAPGEIIGSKGHVTAMKFNGRD-------NESE---LTMKADQVVFAIG 353<br>308 DFLPLVAKSSKAGMCACHSPLPSIRGAVIVLGAGDTAFDCATSALRCGARRVFLVFRKGFVNIRAVPEEVELAKEEKCEFLP<br>FL---SPRKVIVKGGRIVAVQFVRTEQDETGKWNEDEDQIVHLKADVVISAFG 439
Domain Ⅳ (5-FU,FMN)	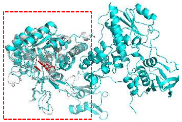	RMSD = 0.619 (255 to 255 atoms)	6 DLSIDFLGVKCENPFFLSSSPVGSNYEMCAKALEAGWGGIYYKTIGIFI--PDECSPRFDITTKEGTPWVG----FKNMEQI<br>SDKPLEVNLEYMRRLKQDYPNKVIVASIMGSN-DEEWTTLAKAVTEAGVDMIECNFSCPQMTSHAMGS-DVGQNPELVKHYCEVVT<br>AATHLPVIAKMTPNIGNMEIPAIASMEGGAAGIAAINTVKAITNIDIENITAMPVVNG--KSSISGYSGAAVKPIALRFITQMKQH<br>PDLVDVPITGVGGIETWRDALEFILVGASNLQVTTAVMQYGYRIVEDMISGLSHYMEDN 308<br>532 DISVEMAGLKFINPFGLASAAPTSSSMIRRAFEAGWGFALTKTFSLDKDIVTNVSPRIVRGTTSGPMYGPGQSSFLNIELI<br>SEKTAAYWCQSVTELKADFPDNIVIASIMCSYNKNDWMELSRKAEASGADALELNLSAPHGMGERGMGLACGQDPELVRNICRWVR<br>QAVQIPFFAKLTPNVTDIVSIARAAKEGGADGVTATNTVSGLMGLKADGTPW-PAVGAGKRTTYGGVSGTAIRPIALRAVTTIARA<br>--LPGFPILATGGIDSAESGLQFLHSGASVLQVCSAVQNQDFTVIQDYCTGLKALLYLK 841
Domain Ⅴ (cFeS1,cFeS2)	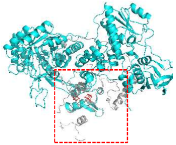	RMSD = 1.023 (79 to 79 atoms)	313 LSDLVGLALPNIVPAEDLDRSFKLLPKFDEDACAGCGRCYVSCFDGGHQAIDWDEEARRPRLNTDKCVGCHLCLNVCPVMDC<br>I 395<br>921 IKDVIGKALQYLGTFGELSNIEQVVAVIDEEMCINCGKCYMTCNDSGYQAIQFDPETHLPTVTD-TCTGCTLCLSVCPIIDC<br>I 1002
